# 

**Published:** 1901-04

**Authors:** 


### Brazos Valley Medical Association.

EASTERLY, TEXAS, March 25, 1901.

The Brazos Valley Medical Association will hold its eleventh semi-annual meeting at Calvert, Texas, the second Tuesday and Wednesday in May next. A full attendance is desired as election of officers will take place at that time.

W. B. BRIGGS, Secretary.

---

### The American Congress of Tuberculosis.

OFFICE OF THE SECRETARY, 39 BROADWAY, N. Y.
February 20, 1901.

DEAR COLLEAGUE: You are respectfully invited to enroll as a member of the American Congress of Tuberculosis, and to contribute a paper to be read at the session, commencing on the 15th day of May and continuing on the 17th day of May, 1901. Please send the title of your paper in advance, as early as possible, and let the paper follow as early as possible, and before April 15th, proximo. Enrolling fee $3.00, entitling you to Bulletin free.

Faithfully yours,

A. N. BELL, M. D., President.

CLARK BELL, Esq., Secretary.

